# Profiling tear film enzymes reveals major metabolic pathways involved in the homeostasis of the ocular surface

**DOI:** 10.1038/s41598-023-42104-2

**Published:** 2023-09-14

**Authors:** Murat Akkurt Arslan, Françoise Brignole-Baudouin, Solenne Chardonnet, Cédric Pionneau, Frédéric Blond, Christophe Baudouin, Karima Kessal

**Affiliations:** 1 Institut National de la Santé et de la Recherche Médicale INSERM UMRS 968, CNRS UMR 7210, Institut de la Vision, IHU ForeSight, Sorbonne Université UM80, 75012 Paris, France; 2 Hôpital National de la Vision des 15-20, INSERM-DGOS CIC 1423, IHU ForeSight, 75012 Paris, France; 3 Hôpital National de la Vision des 15-20, Laboratoire d’Ophtalmobiologie, 75012 Paris, France; 4 Faculté de Pharmacie de Paris, Université de Paris Cité, 75006 Paris, France; 5 INSERM, UMS Production et Analyse des donnees en Sciences de la vie et en Santé, PASS, Plateforme Post-génomique de la Pitié-Salpêtrière, P3S, Sorbonne Université, 75013 Paris, France; 6 Ambroise Paré, Assistance Publique-Hôpitaux de Paris APHP, Service d’Ophtalmologie, Université Versailles Saint-Quentin-en-Yvelines, 92100 Boulogne, France

**Keywords:** Biochemistry, Cell biology, Chemical biology, Medical research

## Abstract

The ocular surface (OS) enzymes are of great interest due to their potential for novel ocular drug development. We aimed first to profile and classify the enzymes of the OS to describe major biological processes and pathways that are involved in the maintenance of homeostasis. Second, we aimed to compare the enzymatic profiles between the two most common tear collection methods, capillary tubes (CT) and Schirmer strips (ScS). A comprehensive tear proteomic dataset was generated by pooling all enzymes identified from nine tear proteomic analyses of healthy subjects using mass spectrometry. In these studies, tear fluid was collected using CT (n = 4), ScS (n = 4) or both collection methods (n = 1). Classification and functional analysis of the enzymes was performed using a combination of bioinformatic tools. The dataset generated identified 1010 enzymes. The most representative classes were hydrolases (EC 3) and transferases (EC 2). Phosphotransferases, esterases and peptidases were the most represented subclasses. A large portion of the identified enzymes was common to both collection methods (n = 499). More enzymes were specifically detected in the ScS-extracted proteome. The major pathways in which the identified enzymes participate are related to the immune system and protein, carbohydrate and lipid metabolism. Metabolic processes for nucleosides, cellular amides, sugars and sulfur compounds constituted the most enriched biological processes. Knowledge of these molecules highly susceptible to pharmacological manipulation might help to predict the metabolism of ophthalmic medications and develop novel prodrug strategies as well as new drug delivery systems. Combining such extensive knowledge of the OS enzymes with new analytical approaches and techniques might create new prospects for understanding, predicting and manipulating the metabolism of ocular pharmaceuticals. Our study reports new, essential data on OS enzymes while also comparing the enzyme profiles obtained via the two most popular methods of tear collection, capillary tubes and Schirmer strips.

## Introduction

Enzymes, the largest group of proteins, are biocatalysts with the critical task of lowering the activation energies of chemical reactions, thus speeding up biochemical reactions in living cells and organisms^1–3^. Diastase was the first enzyme discovered in 1833, and the discovery of other hydrolytic enzymes, such as pepsin and invertase, followed rapidly. The German physiologist, Wilhelm Kühne, first used the term “enzyme” in 1878, stemming from the Greek words *en* (within) and *zumê* (yeast), to describe the ability of yeast to produce alcohol from sugars^1^. In 1836, Swedish chemist, Jöns Jacob Berzelius, introduced the concept of catalysts—chemicals facilitating a reaction without undergoing any change themselves^4^. Enzymes are highly specific to the reactions they catalyze due to the specific binding of substrates to their active sites^2^. However, some enzymes, known as apoenzymes, are not initially active, and they need to be activated by a cofactor. Cofactors can consist of molecules such as metal ions (e.g., Zn) or organic compounds and can bind either covalently or noncovalently with the apoenzyme. The complex formed by a cofactor and apoenzyme is known as a holoenzyme^5^.

The International Union of Biochemistry and Molecular Biology (IUBMB) has standardized enzyme nomenclature based on the Enzyme Commission (EC) system, recommending that enzyme names and classifications should indicate substrates and types of reactions catalyzed^6–9^. Enzymes are classified into seven groups according to the reactions they catalyze: oxidoreductases (EC 1), transferases (EC 2), hydrolases (EC 3), lyases (EC 4), isomerases (EC 5), ligases (EC 6), and translocases (EC 7)^3,10^. The EC number is a four-digit code identifying the enzyme by the reaction catalyzed^11^. The first digit of the EC represents the class of enzyme based on its enzymatic reaction, the second represents the chemical bonds/groups upon which the enzyme acts, the third represents the sub-subclass, and the fourth denotes the specific enzyme within its sub-subclass^12^. The various enzyme classes and the corresponding reactions they catalyze are shown in Table 1.

**Table 1:** Enzyme classes and the reactions they catalyze.

Classification	Class name	Reaction(s) catalyzed	Reactions
EC 1	Oxidoreductases	Catalyze oxidoreductions	XH + Y → X + YH (reduction) or A + O → AO (oxidization)
EC 2	Transferases	Transfer a group (e.g., methyl or glycosyl group) from one compound to another	XY + Z → X + YZ
EC 3	Hydrolases	Catalyze the hydrolysis of various bonds	XY + H_2_O → XOH + YH
EC 4	Lyases	Cleave C–C, C-O, C-N and other bonds by means other than hydrolysis or oxidation	RCOCOOH → RCOH + CO_2_ or [A–X + Y-B] → [X = Y + A-B]
EC 5	Isomerases	Catalyze changes within a single molecule	XYZ → YZX
EC 6	Ligases	Catalyze the joining of two molecules or two parts of a molecule in the presence of ATP	X + Y + ATP → XY + ADP + Pi
EC 7	Translocases	Catalyze the movement of ions or molecules across membranes or their separation within membranes	X_(membrane side 1)_ → X_(membrane side 2)_

Seven classes of enzymes and their catalyzed reactions are shown. Enzymes of the different classes react on different chemical bonds to catalyze the reactions. Data was collected from the Enzyme Database^7^. The various molecules involved in these reactions are represented by A, B, R, X, Y and Z.

Bodily fluids contain a large number of enzymes which play important roles in numerous functions such as metabolism, cell division, gene expression, and immune system reactions^3^. As a bodily fluid, the tear film (TF), which covers the ocular surface (OS) and forms a barrier between the eye and the external environment, is no exception^13–15^. Indeed, homeostasis of the TF is maintained by specific enzymes, which are produced by the lacrimal glands and epithelial cells of the cornea and conjunctiva^16^. The TF plays an essential role in maintaining homeostasis by protecting, nourishing, and lubricating the OS^17,18^. Enzymes are crucial components of the TF, and dysregulation of their expression or activity can result in OS disorders, such as dry eye disease^16,19^.

Many TF enzymes are protective, playing significant roles in antioxidative defense and cell turnover^20^. The two most abundant TF enzymes, lysozyme and lactoferrin, are key antimicrobial components of the OS immune system^20–22^. TF enzymes are so critical to OS homeostasis that simply quantifying their expression and activity in an individual can provide important information regarding the overall health status of the OS. Thus, enzymatic activities present on the OS are of major interest, so as to fully decipher the role of enzymes in the defense and homeostasis of the OS.

The ever-evolving technology of mass spectrometry has enabled the identification of over one thousand proteins in the TF across several proteomic studies^23–25^. Various open-access databases exist to identify and classify enzymes and provide information on their biochemical properties (e.g., ExplorEnz, IntEnz, SIB-ENZYME and BRENDA) from previously generated proteomic datasets^26,27^. More recently, our team and, to the best of our knowledge, only one other study had described the complete profile of TF enzymes in their proteomic data^23,28^. Some other studies have only referred to enzyme groups rather than profiling all enzymes detected^29,30^. In the present study, we merge our prior tear proteomic data with additional comprehensive proteomic analyses identifying a large number of proteins in the TF of healthy subjects by mass spectrometry to more extensively describe the profile of the OS enzymes. This study also aimed to compare the profiles of these enzymes between the two most common tear collection methods, capillary tubes and Schirmer strips. The large enzyme dataset thus created will enable us to reveal the complete landscape of BPs and pathways involved in OS homeostasis.

## Methods

### Selection of proteomics studies for creation of the dataset

A comprehensive tear enzyme dataset from healthy subjects was generated by combining nine comprehensive TF proteomic studies using mass spectrometry. In these studies, between 450 and 2000 proteins were identified using classical tear collection methods (capillary tube and/or Schirmer strip). Of these nine studies (n = 9), four used ScS (n = 4), four used the capillary method (n = 4), and one used both methods (n = 1). In these studies, the experimental protocols were established in accordance with the ethical guidelines of the Declaration of Helsinki and were approved by the relevant institutional ethics committees. Sample collection was also conducted in accordance with the Declaration of Helsinki, and written informed consent was obtained from all subjects who participated^23,24,30–33^. Some of these studies were conducted with healthy laboratory volunteers^25,28,29^. Our study was approved by the Ethics Committee CPP–Ile-de-France (number: 2018-A02800-55). The details of these studies are shown in Table 2. Here, enzymes identified from studies using the capillary method are considered “TF” enzymes, and those from studies using ScS are considered “ScS extracted proteome (SEP)” enzymes.

**Table 2:** List of proteomics studies used for analytical description of the tear film.

Tear sampling method	Authors, year of publication	Number of healthy subjects, samples, extraction method (only for ScS)	Peptide fractionation	Mass spectrometry technology	Protein identification criteria	Number of proteins identified
Capillary tube	De Souza et al., Genome Biol., 2006^29^	Pooled samples (from one subject)	pre-fractionation of proteins with 1D SDS-PAGE (13 fractions) or in-solution digestion of whole samples (without fractionation)	LTQ-FT and LTQ-Orbitrap	Two peptides with Mascot scores of > 35, Two peptides with Mascot scores of > 27 (*p* ≤ 0.01), or one peptide with a Mascot score of > 54 (*p* ≤ 0.0001), when MS3 was performed	491
	Kandhavelu et al., J. Proteomics, 2016^31^	Pooled samples (from 9 subjects)	1D SDS-PAGE pre-fractionation of proteins (26 fractions) or N-linked glycoprotein enrichment	LTQ-Orbitrap Velos Pro	One unique peptide, FDR < 5%	1873
	Hua et al., BMC Ophthalmol.*,* 2020^32^	3 samples (from 3 subjects)	In-solution digestion of whole samples (no pre-fractionation)	LTQ-Orbitrap XL	FDR < 1%	949
	Nättinen et al., Trans. Vis. Sci. Tech., 2020^30^	31 samples (from 31 subjects)	In-solution digestion of whole samples (no pre-fractionation)	TripleTOF 5600 + in SWATH-MS mode	FDR < 1%	404
	Ponzini et al., Int. J. Mol. Sci., 2021^33^	23 samples (from 23 subjects)	In-solution digestion of whole samples (no pre-fractionation)	Orbitrap fusion	One unique peptide, FDR of < 1%	932
Schirmer strip	Zhou et al., J. Proteomics, 2012^24^	4 ScS (from 4 subjects), samples extracted in 100 mM AmBic, at room temperature for 3 h	Offline SCX fractionation of peptides (6 fractions)	TripleTOF 5600	FDR < 1% for peptides	1543
	Aass et al., Anal. Biochem., 2015^25^	48 ScS samples (from 3 subjects) extracted in 100 mM AmBic, NaCl, a surfactant, or a combination of them at 25 °C for 4 h	Offline SCX fractionation of peptides (16 fractions)	LTQ-Orbitrap XL hybrid	Peptide and protein with FDRs of < 1% (high) and 5% (relaxed) LTQ-Orbitrap	1526
	Dor et al., Exp. Eye Res., 2019^23^	16 ScS samples (from 8 subjects) centrifuged at 7840 g for 7 min at 4 °C with no additional buffer	Off-gel electrophoresis of peptides (12 fractions)	LTQ-Orbitrap Velos Pro TripleTOF 5600 + in SWATH-MS mode	Two unique peptides, FDR < 1%	1351
	Nättinen et al., Trans. Vis. Sci. Tech., 2020^30^	31 ScS samples (from 31 subjects) incubated in 50 mM AmBic on ice for 60 min	In-solution digestion of whole samples (no pre-fractionation)	TripleTOF 5600 + in SWATH-MS mode	FDR < 1%	908
	Akkurt Arslan et al. Metabolites, 2021^28^	8 ScS samples (from 2 subjects) extracted in 100 mM ammonium bicarbonate at 4 °C for 4 h	In-solution digestion of whole samples (no pre-fractionation)	TimsTOF Pro	FDR < 1%	1582

The studies included for the analysis of enzymes in this study collected tear samples using the capillary method or Schirmer strips. These studies report comprehensive datasets of the healthy human tear proteome, and all of them used mass spectrometry.

*FDR* false discovery rate, *SCX* strong cation exchange, *ScS* Schirmer strips, *AmBic* ammonium bicarbonate.

### Enzyme classification

The enzymes were classified according to the universal nomenclature proposed by the International Union of Biochemistry and Molecular Biology (IUBMB) and the open-access enzyme database, ExplorEnz (https://www.enzyme-database.org/), as the primary source of enzyme dataset query^10^. All of the enzymes identified in the dataset generated were classified by enzyme commission (EC) number. The Universal Protein Resource (UniProt) database (https://www.uniprot.org/, accessed on 21 April 2022), which incorporates the IUBMB enzyme list data into its datasets, was used to describe principal classes and subclasses of the enzymes^34^.

### Functional analysis of the enzyme dataset

Functional analysis of the identified enzymes was performed by combining various free and open-source bioinformatics databases. The analysis of major signaling pathways was performed using Reactome^35^ (https://reactome.org/PathwayBrowser/#/, accessed on July 29, 2022) and the Kyoto encyclopedia of genes and genomes (KEGG)^36^ (https://www.genome.jp/kegg/pathway.html, accessed on August 7, 2022). Functional enrichment analysis of cellular components was conducted using the Database for Annotation, Visualization and Integrated Discovery (DAVID)^37^ (https://david.ncifcrf.gov/, accessed on August 10, 2022), and enrichment analysis of BPs and pathways was performed using Metascape^38^, (https://metascape.org/gp/index.html#/main/step1, accessed on April 21, 2022). Statistical analysis to calculate the enriched terms of GO BPs and pathways was performed with Metascape software using the Benjamini–Hochberg *p-*value correction algorithm and hypergeometric test to identify all ontology terms^38^.

### Ethics approval and consent to participate

The experimental protocols were established in accordance with the ethical guidelines of the Declaration of Helsinki and were approved by the relevant institutional ethics committees. Some subjects were laboratory volunteers; in other cases, written informed consent was obtained from all subjects. The images used to illustrate sample collection were obtained from the laboratory volunteers. Our study was approved by the Ethics Committee CPP–Ile-de-France (number: 2018-A02800-55).

## Results

### Profiling and classification of enzymes in TF and SEP

**Figure 1:**
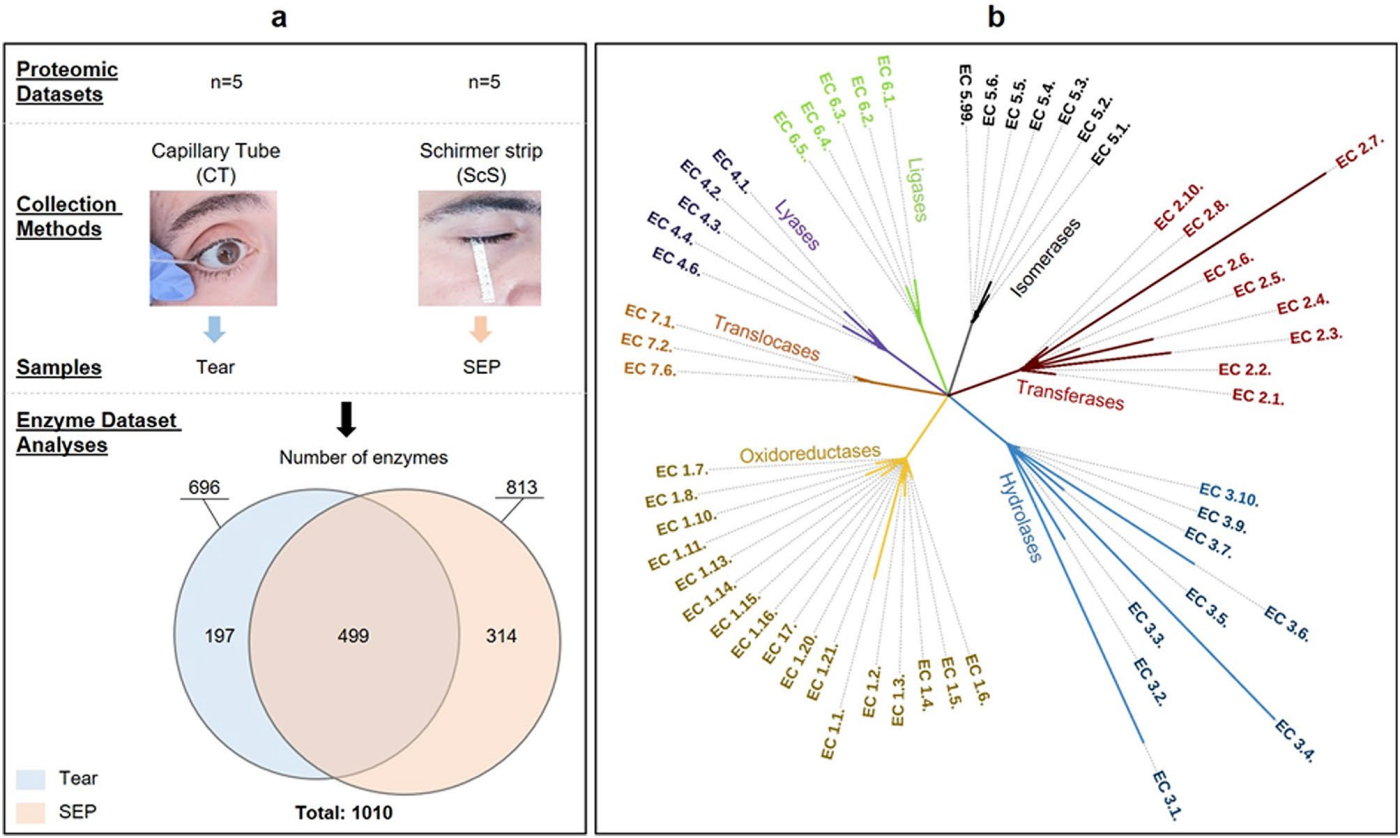
Distribution and classes of identified enzymes according to sample collection method. (**a**) Number of enzymes identified according to tear collection methods. In total, 1010 enzymes were identified, 891 of which were reviewed proteins from UniProtKB/Swiss-Prot, while the others (119) represented unreviewed proteins from UniProtKB/TrEMBL. (**b**) Subclassification of all identified enzymes into the seven enzyme groups. Line length is proportional to the number of enzymes in each subclass.

The comprehensive enzyme dataset generated in this study included 1010 enzymes (Fig. 1a). Analysis of the SEP identified more enzymes (n = 813) than TF analysis (n = 696). Table S1 displays the contribution of each study, indicating the number and percentage of identified enzymes within the complete enzyme dataset.

The list of these compiled enzymes is provided in the “Enzyme Dataset Table in the supplementary data.” The distribution of enzymes compiled from among these two groups (TF and SEP enzymes) revealed a large number of common enzymes (499) and specific enzymes related to each collection method (n = 197 for TF and n = 314 for SEP) (Fig. 1a). All seven classes of enzymes (EC1-7), as outlined by IUBMB enzyme nomenclature, appeared in our analysis (Fig. 1b). Of all identified enzymes, peptidases (EC 3.4, n = 172), phosphotransferases (EC 2.7, n = 156) and esterases (EC 3.1, n = 151) were the most represented subclasses. Within the oxidoreductase class (EC 1), various dehydrogenases (n = 67) (e.g., alcohol and aldehyde dehydrogenases) represented nearly half of the enzymes.

### Comparison of enzyme profiles in TF and SEP

**Figure 2:**
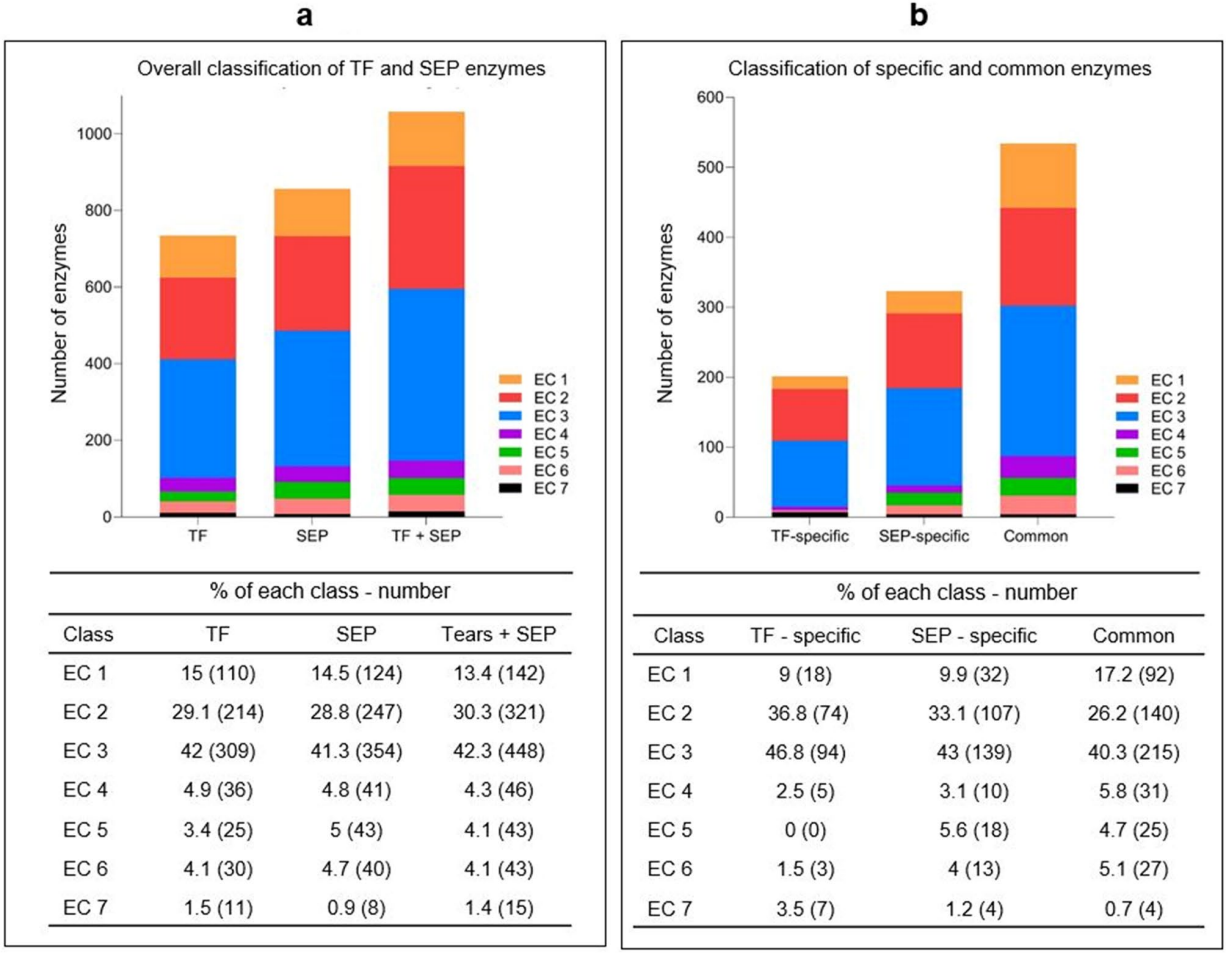
Comparison of enzyme classes in TF and SEP. (**a**) Distribution of enzymes identified in TF, SEP and both methods combined. Number of each class in the TF, SEP and combined methods (TF + SEP), (**b**) Specific and common enzymes in TF and SEP. Number of specific enzymes detected only in TF, only in SEP, and by both methods are shown.

The most represented classes of all the identified enzymes were hydrolases (EC 3, 42.3%) and transferases (EC 2, 30.3%), whereas translocases (EC 7, 1.4%) formed the least represented class (Fig. 2a). The distribution of the seven enzyme classes was largely similar between TF and SEP. However, analyses of the enzymes that were SEP-specific or TF-specific revealed substantial differences between the percentage and number of each enzyme class (Fig. 2b). For instance, isomerases (EC 5) and ligases (EC 6) in the SEP outnumbered these enzymes detected in the TF, both numerically and proportionally. No specific isomerase was identified in TF, while isomerases formed 5.6% of the specific enzymes in the SEP (Fig. 2b).

### Major signaling pathways of TF and SEP enzymes

**Figure 3:**
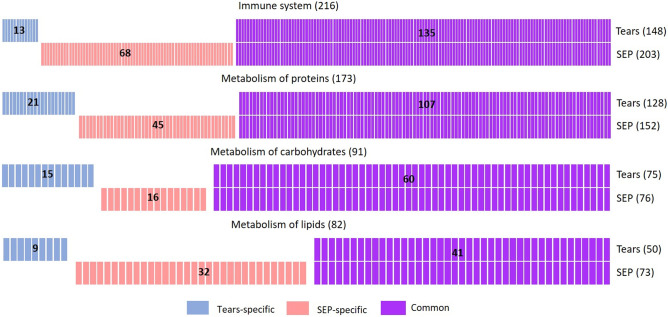
Barcode representation of enzymes in immune and metabolic pathways. Each bar represents one protein. Blue bars represent TF-specific enzymes, pink bars SEP-specific enzymes, and purple bars common enzymes. The number of enzymes is also displayed numerically within each group of bars, and the total number of enzymes involved in each progress is shown in parentheses on the right.

Functional analysis of all the enzymes revealed roles in several signaling pathways. The major signaling pathways of the identified enzymes were related to the immune system and protein, carbohydrate and lipid metabolism (Fig. 3). The largest number of enzymes participate in the immune system and protein metabolism. The majority of the enzymes in these pathways were common amongst the two tear collection methods. In these four main pathways, more enzymes were described in the SEP. However, in the carbohydrate metabolism pathways, almost the same number of enzymes were identified by each collection method. The list of enzymes involved in these pathways is shown in Table S2. A large number of enzymes involved in carbohydrate and protein metabolism were those responsible for glycosylation processes, glycan biosynthesis and metabolism. These enzymes are shown in bold in Table S2.

### Enrichment analysis of TF and SEP enzymes

**Figure 4:**
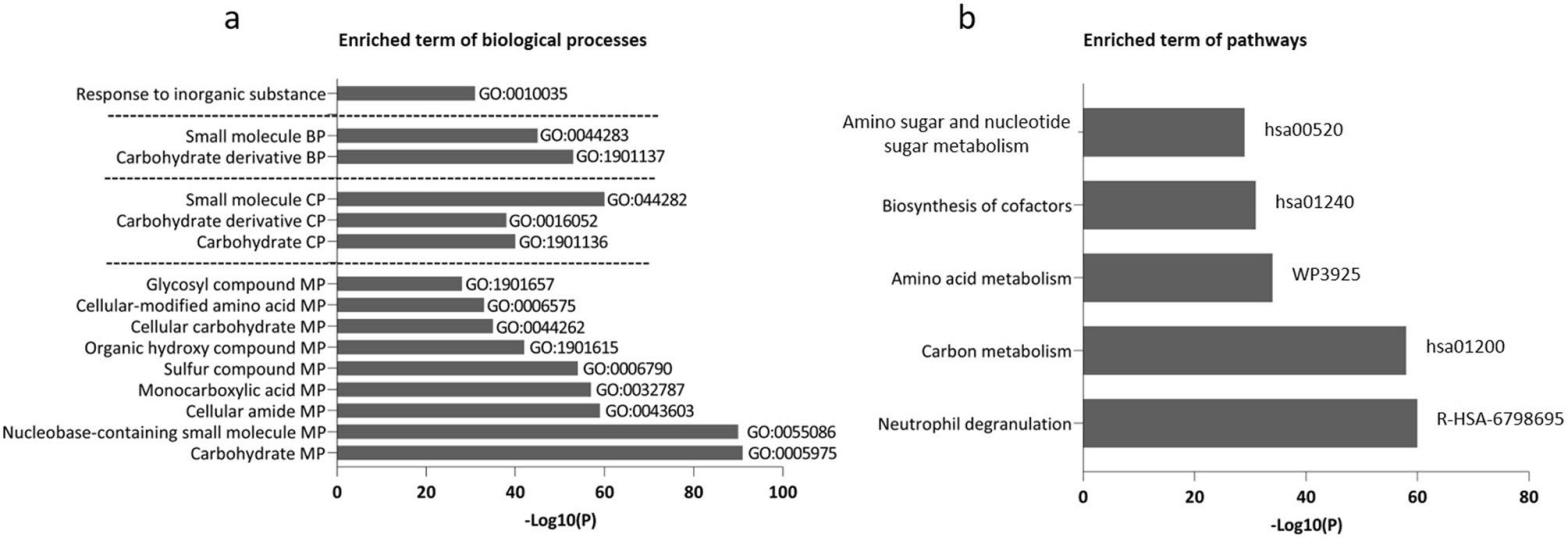
Enriched terms of GO biological processes and signaling pathways. The term “Log10(P)" refers to the p-value in log base 10. MP*, metabolic process, CP**, catabolic process, BP***, biosynthetic process.

The enriched terms of BPs and pathways of all identified enzymes are shown in Fig. 4. Metabolic processes such as those for carbohydrates, nucleosides, cellular amides and glycosyl compounds were among the enriched terms of BPs. Catabolic and biosynthetic processes for carbohydrates and their derivatives constituted other significant terms of BPs. Among pathways, neutrophil degranulation was the most significant pathway. Carbon and amino acid metabolism represented two other highly significant pathways. The details of these enriched terms and the number of enzymes involved in each term are presented in Table S3.

**Figure 5:**
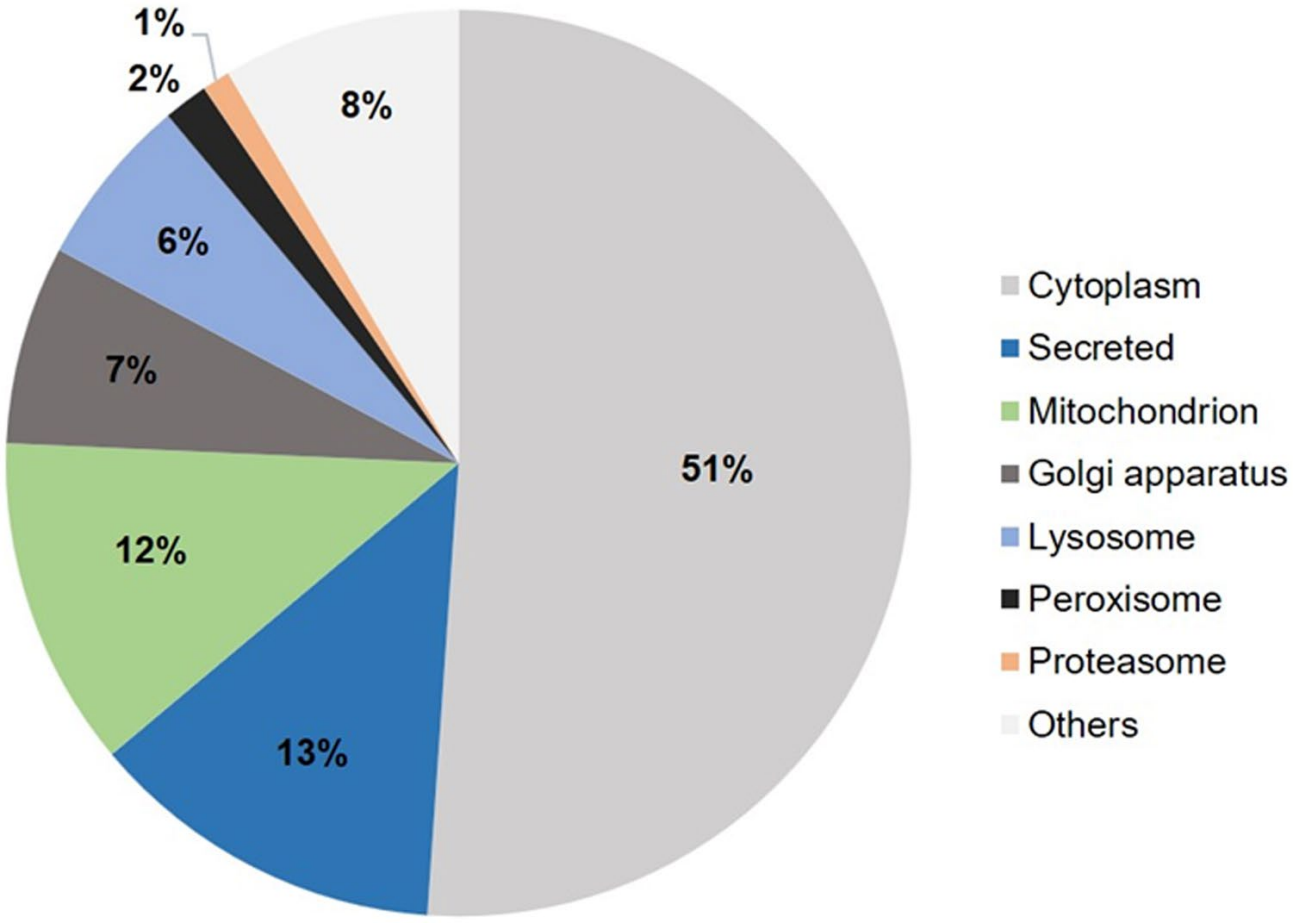
Cellular distribution of all enzymes identified in TF and SEP. The majority of enzymes were localized in the cytoplasm. Data were retrieved using DAVID software, functional annotations—cellular component.

Enrichment analysis of cellular components revealed that over half of all identified enzymes were localized in the cytoplasm. The remaining enzymes were either secreted enzymes (13%) or those localized in cellular organelles such as mitochondria and lysosomes, among others (Fig. 5).

## Discussion

The constant evolution of technologies dedicated to proteomic investigations has enabled the identification of thousands of proteins in numerous studies^28,39,40^. The proteomic dataset for tears has been continuously expanding due to a high number of published proteomics studies^41^. In this large dataset for the tear proteome, particular attention has been paid to enzymes, due to their involvement in numerous critical BPs on the OS. Merging the most comprehensive tear proteomic datasets represents a rational methodology to identify and classify all enzymes detected in TF. Our analysis demonstrates that the TF is as complex as blood in terms of the number of enzymes it contains^42^. Additionally, this analysis enables us to unveil the similarities and differences between the enzyme profiles of tear samples collected by capillary tube (CT) and Schirmer strips (ScS). Upon tear collection, various factors such as storage, extraction, handling, and analytical methods could also influence the biochemical profile of tears^43^. Moreover, it is worth emphasizing that not only the methods of sample collection, but also the demographic characteristics of the subjects, sample processing techniques, and analytical methods employed might have an impact on the outcomes of proteomic studies^44^. The variability in sample collection practices and the absence of standardized pre-analytical methods represent significant challenges to achieving reproducible results and facilitating comparisons across various studies. Therefore, all of these aspects should be considered before choosing the most suitable method, since each sampling method has its own advantages and disadvantages^45^.

It should be noted that ScS can collect conjunctival cells in addition to TF, potentially resulting in a different proteomic profile^30^. Hence, in SEP, more enzymes were detected than from TF. Enrichment analysis of the cellular compartments also revealed that the percentage of cytoplasmic enzymes was higher among SEP enzymes with 54% vs 49.6%, while secreted enzymes were proportionately more abundant in TF with 14.6% vs 12%.

**Figure 6:**
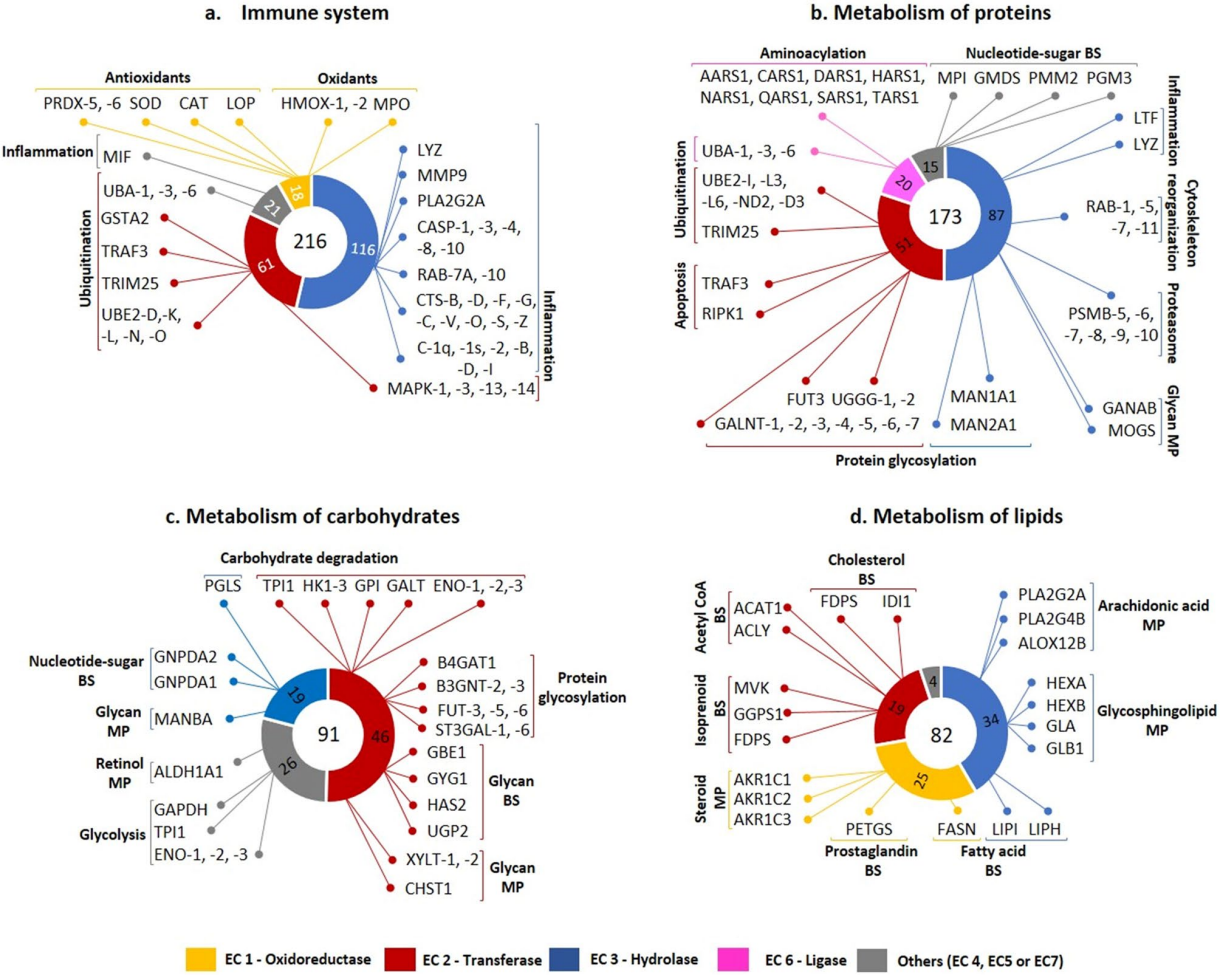
Identification of enzymes involved in four major signaling pathways. Some examples of enzymes, along with their group name and function, are shown in the pathways of (**a**) the immune system, (**b**) protein metabolism, (**c**) carbohydrate metabolism and (**d**) lipid metabolism. *BS* biosynthesis, *MP* metabolic process.

The other portion of this study sheds light on BPs and pathways describing the potential roles of these enzymes. The majority of enzymes identified play a role in the immune system. Indeed, TF is the first line of defense for the eye and, therefore, contains numerous antibacterial molecules and enzymes^17^. The contribution of the antimicrobial function of TF to the immune response has long been recognized^46^. Our study revealed that the immune system enzymes identified are made up primarily of hydrolases and transferases. Examples of these enzymes are shown in Fig. 6a. A large number of enzymes take part in the neutrophil degradation pathway. This pathway is a known component of the innate immune defense mechanisms of the OS.

Increased TF osmolarity activates mitogen-activated protein kinases (MAPKs), matrix metalloproteinases (MMPs) and those involved in lipid metabolism such as sphingomyelinase 2^47,48^. Activation of MAPKs increases the activity of MMPs, which play roles in inflammation, tissue remodeling and degradation of the extracellular matrix^49^. Elevated tear osmolarity might increase the blink reflex, generating shear stress on the OS while spreading the tear fluid^50,51^. Our analysis identified some of these enzymes (e.g., MAPK-13, -14, MMP9, CTSL, RAC1 and GST) involved in the shear stress pathway.

The majority of enzymes identified participate in the immune system and protein, lipid, and carbohydrate metabolism. Some examples of these enzymes are shown in Fig. 6. Some of the identified oxidative and antioxidative enzymes also take part in the immune system (Fig. 6a). Antioxidant enzymes on the OS provide a sophisticated level of protection to the eye against oxidative stress^52,53^. Products of reactive oxygen species (ROS) have important roles in homeostasis and cell signaling, yet an imbalance of ROS production or scavenging causes oxidative damage resulting in cellular damage and death^54,55^. OS tissues, such as the cornea, possess mechanisms protective against endogenous oxidative stress^56^. Mitochondria, peroxisomes and microsomes are generators of endogenous ROS. Additionally, enzymes such as membrane-associated NADPH oxidase, cytochrome c oxidase, and xanthine oxidase are other endogenous sources of ROS^57^. The most studied antioxidant enzymes of the OS are catalase (CAT), glutathione peroxidase (GPx), superoxide dismutase (SOD) and peroxiredoxins^53,56^. In our analysis, the most important antioxidant enzymes, such as GPx1-3-4, SOD1-2-3, CAT and peroxiredoxins (PRDX1-2-3-4-5-6), were identified. On the other hand, oxidase enzymes, such as cytochrome c oxidases (COX1-2-3), heme oxygenases (HMOX1-2) and sulfhydryl oxygenase (QSOX1), were also detected.

Enzymes responsible for protein metabolism play roles mainly in the pathways of protein biochemical modification, such as ubiquitination, glycosylation and aminoacylation (Fig. 6b). These post-translational modifications are essential for the proper function of the proteins^58^. Among the proteins that participate in protein and carbohydrate metabolism, a large number of enzymes identified were responsible for glycosylation and glycan metabolism, which highlights significant biosynthesis and metabolism of the glycocalyx, mucin and nucleotide-sugar biosynthesis on the OS (Fig. 6b, c). The possible role of glycosylation in homeostasis of the OS has been accepted for quite some time, since glycosylated mucins, aminoglycans and sphingolipids are very abundant on the OS^59^. Indeed, enzymes responsible for fucosylation (a type of glycosylation), such as FUT-3, -5, and -6, regulate inflammation. It has been shown that FUT1 knockout (KO) mice developed corneal epithelial defects and stromal opacity under desiccative stress, while corneal integrity was not altered and stromal opacity was less prominent in the wild-type. Meibomian gland function was also altered in these KO mice^59,60^. Apart from enzymes involved in glycoprotein metabolism, various enzymes, such as HEXA, HEXB (glycosidases) and GLA, GLB1 (galactosidases), involved in glycosphingolipid metabolism appeared in our analysis (Fig. 6d).

Delving further into the role of glycosylation in OS homeostasis, heavily glycosylated transmembrane mucins, Mucin-1, -4 and -16 and the gel-forming mucin (Mucin 5AC), are crucial components of the TF that mediate signal transduction, lubrication, and hydration to protect the OS from pathogens and mechanical or chemical damage. The composition of mucins is subjected to enzymatic modifications for a stable defense^61–63^. These enzymes possess a central role in maintaining glycocalyx structure and mucin production, which are critical for wettability of the OS and viscosity of the TF^46,64,65^.

Initial focus on mucins as the main contributors to tear film's non-Newtonian behavior was challenged by low mucin concentrations found in quantification studies. This highlights the need for a deeper understanding of tear component concentrations. While certain proteins such as lysozyme, secretory immunoglobulin A, lactoferrin, albumin, immunoglobulin G, and lipocalin were initially thought to have limited impact on viscosity, interactions between different proteins and lipids have shown significant influence on tear viscosity, emphasizing the complex interplay of tear components^66^. The shear stress pathway could have a role in the control of tear viscosity and volume. A dysregulation among tear components that contribute to tear viscosity and shear stress, might affect the activity of enzymes involved in this pathway. Therefore, these enzymes might be potential therapeutic targets. Among these enzymes, MAPKs, MMP9, IKBKB and AKT1 are also involved in the TNFα signaling pathway and MAPK13, -14, MMP9, IKBKB, and HSP90AA1 participate in the IL-17 signaling pathway. Our results might highlight the role of these enzymes in the control of OS rheology and their involvement in the inflammation of the OS in the case of increased shear stress. The lipidome of the OS cells also is disrupted by increased TF osmolarity^67^. In the presence of all of these changes, the expression and activity of enzymes responsible for these metabolic activities could be modulated.

Glycosyltransferases, glycosidases and glycan-modifying enzymes are responsible for the biosynthesis of mucin-type O-glycans and N-glycans on the OS^68^. The mucin-type O-glycosylation, which provides mucins with viscoelastic properties, is one of the most abundant forms of protein glycosylation, forming more than 70% of the mass of mucins^63,69,70^. The attachment of glycans to serine and threonine residues via O-linked N-acetylgalactosamine (GalNAc) is controlled by polypeptide GalNAc-transferases (GalNAc-Ts)^69^. The GalNAc-Ts initiate the biosynthesis of mucin-type O-glycans in the Golgi apparatus^38^. For instance, O-glycosylation of Mucin 2 occurs post-translationally by adding GalNAc to the hydroxyl groups of serine and threonine in mice^71^. In the current study, seven GalNAc-Ts were identified.

GalNAc-T4 is present in the apical, and GalNAc-T2 in the basal, cell layers, whereas GalNAc-T6 is restricted exclusively to conjunctival goblet cells^72^. These enzymes take part in the synthesis of N-glycans (glycoproteins), such as lacritin, clusterin, lactoferrin, and secretory IgA, which are associated with pathogen adhesion and elimination in the tears^68,73^. Glycan synthesis in the TF may be disrupted in disease states altering the integrity of epithelial cells and the regular function of transmembrane mucins^74^. Indeed, down-regulation of mucin O-glycosylation by knockdown of C1GALT1 (T-synthase) decreases mucosal barrier function and increases epithelial permeability^61^. Inflammation also alters the O-glycosylation process in corneal and conjunctival epithelial cells^75^. Various genes associated with glycan synthesis, including mucin-type glycosyl-transferases, are likely significantly dysregulated in dry eye disease, an inflammatory disease of the OS^62,76^. Our dataset reliably detected and provides further information regarding the role of enzymes in mucin and glycocalyx homeostasis on the OS.

The eye also possesses a complex metabolic system to prevent entry of xenobiotics from both the environment and systemic circulation into the ocular tissues^77,78^. This ocular metabolism plays an important role in the pharmacokinetics of topical medications. However, drug-metabolizing enzymes in the eye have not yet been entirely characterized^79–81^. A significant knowledge gap remains to be filled, as the OS may perform some organ-specific metabolism, distinct from the well-known hepatic metabolism^79^. Ocular tissues contain numerous enzymes responsible for the metabolism of medications and other xenobiotics, including oxidoreductases (e.g., cyclooxygenase, cytochrome p450), hydrolases (e.g., aminopeptidase, carboxyl esterase, phosphatase, β-glucuronidase) and transferases (e.g., arylamine acetyltransferase, glutathione S-transferase)^80^. In corneal tissues, most of these enzyme classes participate in drug metabolism^56^. These enzymes play roles at different stages of drug metabolism. Oxidases, reductases and hydrolyses are involved in phase I, and conjugating enzymes are involved in phase II drug metabolism to convert these substances into large water-soluble metabolites. In phase III, the metabolite is further metabolized and finally excreted^82^. In our analysis, of the oxidative cytochrome enzyme family, cytochrome c oxidase subunits 1, -2, -3, cytochrome b-561, NADH-cytochrome b5 reductases 2, -3, and NADPH-cytochrome P450 reductase were detected. With regard to glutathione S-transferase (GST), one of the most detoxifying enzymes, its 10 subtypes, designated GST—A1, -A2, -Kappa 1, -LANCL1, -Mu 1, -Mu 2, -Mu 5, -Omega-1, -P, and -theta-1, were identified in our analysis. A large number of identified enzymes, mainly transferases, were involved in drug pharmacokinetics. The majority of these enzymes are expressed in the liver. These enzymes are involved in conjugating molecules such as glutathione, glycine-N-acetyl or sulfo groups to the drugs to facilitate their elimination.

Drug-metabolizing enzymes in the eye can be targeted to activate ophthalmic prodrugs^80^. Ocular esterases are used in the design of prodrugs and the degradation of polymer-based drug delivery systems^83,84^. We identified over one hundred esterases, a large portion of which are involved in metabolism, particularly lipid metabolism. Of these enzymes, liver carboxylesterase 1 and paraoxonase/arylesterase 1 play roles in the pharmacokinetics of medications such as aspirin and atorvastatin. Knowledge of these esterases, together with other drug-metabolizing enzymes, can broaden the range of options for the development of diverse drug delivery systems and new prodrug strategies to prevent drugs from converting too quickly into inactive metabolites^56^. Ocular prodrugs are used mainly to increase permeability through the cornea^83^. Thus, prodrugs of adrenaline (dipivefrin), phenylephrine (phenylephrine oxazolidine) and prostaglandin F2 alpha (latanoprost) are marketed to increase bioavailability and reduce side effects of these ophthalmic medications^83,85,86^. The prodrug form of latanoprost is latanoprost isopropyl ester, which is more lipophilic than latanoprost, and corneal esterases hydrolyze this prodrug into its active acidic form^87^. Another very important feature of enzymes is their high susceptibility to pharmaceutical manipulation^88^. Indeed, the majority of the Food and Drug Administration (FDA)-approved target drugs are designed to bind to enzymes^89^. Compared to other proteins, the high susceptibility of enzymes such as MAPK1, MAPK3, and Caspase-3 to pharmaceutical modulation makes them attractive molecules to be targeted when involved in an ocular disease process. Increased expression and activity of these enzymes during the disease state can be targeted. Comprehensive knowledge of these enzymes might provide the opportunity to improve the design and formulation of ophthalmic medications.

## Conclusions

A wide variety of enzymes exist on the OS, maintaining homeostasis and protecting the OS from external insults. Until now, OS enzymes had not been thoroughly catalogued. This study aimed to fill this significant knowledge gap by generating an advanced dataset that might be helpful in the development of novel ocular prodrugs and targeted drug delivery systems. The large number of enzymes plays a vital role in the homeostasis of the tear mucins and glycocalyx within the ocular surface. These enzymes’ high susceptibility to pharmaceutical manipulation makes them potential therapeutic targets. The development of safer, more effective ophthalmic medications might be facilitated by understanding the mechanisms of enzymes on the OS. Combining the knowledge of OS enzymes with new approaches and techniques might open up new avenues for the development of novel pharmaceuticals and formulations.

### Supplementary Information

Supplementary Information 1.

Supplementary Information 2.

## Data Availability

All data generated or analyzed during this study are included in this published article [Enzyme Dataset Table, in the supplementary data].

## Abbreviations

BPs: Biological processes
CAT: Catalase
CT: Capillary tube
COX: Cytochrome c oxidases
DAVID: Database for Annotation, Visualization and Integrated Discovery
EC: Enzyme Commission
FDA: Food and Drug Administration
OS: Ocular surface
GalNAc: N-acetyl galactosamine
GalNAc-Ts: N-acetyl galactosamine-transferases
GPx: Glutathione peroxidase
GST: Glutathione S-transferase
HMOX: Heme oxygenase
IUBMB: International Union of Biochemistry and Molecular Biology
KEGG: Kyoto Encyclopedia of Genes and Genomes
MAPKs: Mitogen-activated protein kinases
MMPs: Matrix metalloproteinases
PRDX: Peroxiredoxin
QSOX1: Sulfhydryl oxygenase
ROS: Reactive oxygen species
ScS: Schirmer strip
SEP: Schirmer strip-extracted proteome
SOD: Superoxide dismutase
TF: Tear film
