# Supplementary material for: Profiling tear film enzymes reveals major metabolic pathways involved in the homeostasis of the ocular surface
Source: Sci Rep. 2023 Sep 14;13:15231. doi: 10.1038/s41598-023-42104-2 (PMC10502076; doi:10.1038/s41598-023-42104-2)
Supplement: Supplementary file 1 — Supplementary Information 1. [file 41598_2023_42104_MOESM1_ESM.docx]

**Supplementary data**

**Table of enzyme dataset.** List of identified enzymes in tears and Schimer strip-extracted proteome (SEP). Protein ID, protein name, gene name and enzyme commission (EC) number of Tears-specific (n=197), SEP-specific (n=314), and common (n=499) enzymes are provided in this supplementary data. Reviewed proteins in these lists come from the Swiss-Prot section and unreviewed proteins from the TrEMBL section of UniProtKB.

**Table S1.** Number and percentage of identified enzymes in each study within the complete enzyme dataset.

**Table S2.** List of proteins that are involved in the immune system, protein-carbohydrates-lipid metabolism and glycosylation pathways. The enzymes which are additionally involved in glycosylation processes, glycan biosynthesis or metabolic pathways are indicated in bold. The total number of enzymes in each pathway is shown between the brackets.

**Table S3.** Enriched terms of GO biological processes and pathways with their representative enriched terms. The “number” of genes in the user-provided lists with membership in the given ontology term is provided for each term. The percentage of all of the user-provided genes that are found in the given ontology term is shown by "%" (only input genes with at least one ontology term annotation are included in the calculation). The p-value in log base 10 is indicated by "Log10(P)". the multi-test adjusted p-value in log base 10 is shown by "Log10(q)".

**Table S1**

| Tear sampling method | Authors, year of publication | Number and percentage of identified enzymes |
| --- | --- | --- |
| Capillary tube | De Souza *et al*., Genome Biol., 2006 | 178 (17.6%) |
|  | Kandhavelu *et al*., J. Proteomics, 2016 | 244 (24.2%) |
|  | Hua *et al*., BMC Ophthalmol.*,* 2020 | 375 (37.1%) |
|  | Nättinen *et al*., Trans. Vis. Sci. Tech., 2020 | 92 (9.1%) |
|  | Ponzini *et al*., Int. J. Mol. Sci., 2021 | 224 (22.2%) |
| Schirmer strip | Zhou *et al*., J. Proteomics, 2012 | 461 (45.6%) |
|  | Aass *et al*., Anal. Biochem., 2015 | 249 (24.7%) |
|  | Dor *et al*., Exp. Eye Res., 2019 | 507 (50.2%) |
|  | Nättinen *et al*., Trans. Vis. Sci. Tech., 2020 | 297 (29.4%) |
|  | Akkurt Arslan *et al*. Metabolites, 2021 | 531 (52.6%) |

**Table S2**

| Pathway | Gene names | | | | |
| --- | --- | --- | --- | --- | --- |
| Immune system (216) | \| *ACLY* \| \| --- \| \| *ACP3* \| \| *ADAM10* \| \| *AGA* \| \| *AGL* \| \| *AKT1* \| \| *ALAD* \| \| *ALDOA* \| \| *ALDOC* \| \| *ALPK1* \| \| *AMPD3* \| \| *ANPEP* \| \| *APEH* \| \| *APRT* \| \| *ARG1* \| \| *ARIH1* \| \| *ARSA* \| \| *ART1* \| \| *ASAH1* \| \| *ATP6V1A* \| \| ***B4GALT1*** \| \| *BLMH* \| \| *C1R* \| \| *C1S* \| \| *C2* \| \| *CANT1* \| \| *CAPN1* \| \| *CASP1* \| \| *CASP10* \| \| *CASP3* \| \| *CASP4* \| \| *CASP8* \| \| *CAT* \| \| *CBL* \| \| *CDA* \| \| *CDC42* \| \| *CFB* \| \| *CFD* \| \| *CFI* \| \| *CPB2* \| \| *CPPED1* \| \| *CSK* \| \| *CTSA* \| | \| *CTSB* \| \| --- \| \| ***CTSC*** \| \| *CTSD* \| \| *CTSF* \| \| *CTSG* \| \| *CTSH* \| \| *CTSL* \| \| *CTSO* \| \| *CTSS* \| \| *CTSV* \| \| ***CTSZ*** \| \| *CYB5R3* \| \| *DCD* \| \| *DDX3X* \| \| *DDX58* \| \| *DERA* \| \| *DHX9* \| \| *DNM2* \| \| *DPP7* \| \| *DUSP3* \| \| *EIF4A1* \| \| *EIF4A2* \| \| *ELANE* \| \| *ERAP1* \| \| *ERAP2* \| \| *F2* \| \| *FKBP1A* \| \| *FTH1* \| \| ***FUCA1*** \| \| ***FUCA2*** \| \| *FYN* \| \| *GAA* \| \| *GBP1* \| \| *GBP2* \| \| *GBP6* \| \| *GGH* \| \| *GLA* \| \| *GLB1* \| \| *GPI* \| \| *GSTA2* \| \| *GSTO1* \| \| *GSTP1* \| \| *GYG1* \| | \| *HERC4* \| \| --- \| \| ***HEXB*** \| \| *HK3* \| \| *HMOX1* \| \| *HMOX2* \| \| *HSP90AA1* \| \| *HSPA5* \| \| *HSPA8* \| \| *HUWE1* \| \| *IDH1* \| \| *IFI30* \| \| *IKBKB* \| \| *IMPDH2* \| \| *IRAK4* \| \| *ISG20* \| \| *LGMN* \| \| *LPO* \| \| *LTA4H* \| \| *LTF* \| \| *LYN* \| \| *LYZ* \| \| *MAN2B1* \| \| *MANBA* \| \| *MAP2K3* \| \| *MAP2K4* \| \| ***MAPK1*** \| \| *MAPK13* \| \| *MAPK14* \| \| ***MAPK3*** \| \| *MAPKAPK3* \| \| *MASP1* \| \| *MIF* \| \| *MMP8* \| \| *MMP9* \| \| *MPO* \| \| *MTAP* \| \| *NAPRT* \| \| *NEU1* \| \| *NIT2* \| \| *NME2* \| \| *NPEPPS* \| \| *P4HB* \| \| *PAFAH1B2* \| | \| *PAK2* \| \| --- \| \| *PDIA3* \| \| *PDXK* \| \| *PFKL* \| \| *PGAM1* \| \| *PGLYRP2* \| \| *PGM1* \| \| *PGM2* \| \| *PIN1* \| \| *PKM* \| \| *PLA2G2A* \| \| *PNP* \| \| *PPIA* \| \| *PPM1B* \| \| *PPP2CA* \| \| *PPP2CB* \| \| *PPP3CA* \| \| *PRDX4* \| \| *PRDX6* \| \| *PRKCB* \| \| *PRKCD* \| \| *PRTN3* \| \| *PSMB10* \| \| *PSMB5* \| \| *PSMB6* \| \| *PSMB7* \| \| *PSMB8* \| \| *PSMB9* \| \| *PSMD14* \| \| *PTK2* \| \| *PTPN11* \| \| *PTPN13* \| \| *PTPN23* \| \| *PTPN6* \| \| *PTPRJ* \| \| *PYGB* \| \| *PYGL* \| \| *QPCT* \| \| *QSOX1* \| \| *RAB10* \| \| *RAB27A* \| \| *RAB5B* \| \| *RAB5C* \| | \| *RAB7A* \| \| --- \| \| *RAC1* \| \| *RAP1A* \| \| *RAP1B* \| \| *RBX1* \| \| *RHOA* \| \| *RIPK1* \| \| *RNASE3* \| \| *RNASE7* \| \| *RNASET2* \| \| *RNF213* \| \| *RNF216* \| \| *RPS6KA1* \| \| *RPS6KA3* \| \| *SAMHD1* \| \| *SOD1* \| \| *SOD2* \| \| *SRC* \| \| *STUB1* \| \| *TALDO1* \| \| *THOP1* \| \| *TKFC* \| \| *TLR2* \| \| *TPP2* \| \| *TRAF3* \| \| *TRIM25* \| \| *UBA1* \| \| *UBA3* \| \| *UBA6* \| \| *UBE2D2* \| \| *UBE2D3* \| \| *UBE2K* \| \| *UBE2L3* \| \| *UBE2L6* \| \| *UBE2M* \| \| *UBE2N* \| \| *UBE2O* \| \| *UBR4* \| \| *USP14* \| \| *VAT1* \| \| *VCP* \| \| *XDH* \| \| *XRCC5* \| \| *XRCC6* \| |
| Protein metabolism (173) | \| *AARS1* \| \| --- \| \| *ACE* \| \| *ADAM10* \| \| ***ADAMTS20*** \| \| ***ADAMTS6*** \| \| *AGBL1* \| \| *ALPL* \| \| *ANPEP* \| \| *APEH* \| \| *ARSA* \| \| ***B3GNT2*** \| \| ***B3GNT3*** \| \| ***B3GNT7*** \| \| ***B4GALT1*** \| \| ***B4GAT1*** \| \| *CARS1* \| \| *CES1* \| \| *COPS5* \| \| *CP* \| \| *CPB2* \| \| *CPM* \| \| ***CTSA*** \| \| ***CTSC*** \| \| *CTSD* \| \| *CTSG* \| \| *CTSH* \| \| ***CTSZ*** \| \| *DARS1* \| \| *DDX17* \| \| *DDX5* \| \| *DDX58* \| \| *DPP4* \| \| *EIF2S3* \| \| *EIF3F* \| \| *EIF4A1* \| | \| *EIF4A2* \| \| --- \| \| *EIF5B* \| \| *EPRS1* \| \| *ERO1A* \| \| *F2* \| \| *FARSA* \| \| *FARSB* \| \| ***FCSK*** \| \| *FN3KRP* \| \| ***FUCA1*** \| \| ***FUCA2*** \| \| *FURIN* \| \| ***FUT3*** \| \| ***GALNT1*** \| \| ***GALNT2*** \| \| ***GALNT3*** \| \| ***GALNT4*** \| \| ***GALNT5*** \| \| ***GALNT6*** \| \| ***GALNT7*** \| \| ***GANAB*** \| \| *GAPDHS* \| \| *GARS1* \| \| *GBA* \| \| ***GCNT3*** \| \| ***GFPT1*** \| \| ***GFUS*** \| \| *GGCX* \| \| ***GLB1*** \| \| ***GMDS*** \| \| ***GMPPB*** \| \| ***GNPNAT1*** \| \| *GPLD1* \| \| *HARS1* \| \| *HDAC4* \| | \| *HSPA8* \| \| --- \| \| *IARS1* \| \| *IDE* \| \| *KARS1* \| \| *KLK13* \| \| *LTF* \| \| *LYZ* \| \| ***MAN1A1*** \| \| ***MAN2A1*** \| \| ***MGAT1*** \| \| ***MGAT2*** \| \| ***MGAT5*** \| \| ***MOGS*** \| \| ***MPI*** \| \| *MSRA* \| \| ***NAGK*** \| \| ***NANS*** \| \| *NAPSA* \| \| *NARS1* \| \| ***NEU1*** \| \| ***NEU2*** \| \| ***NSF*** \| \| *OTUB1* \| \| *P4HB* \| \| *PARK7* \| \| *PCMT1* \| \| *PCSK2* \| \| ***PDIA3*** \| \| *PDIA6* \| \| ***PGM3*** \| \| *PLG* \| \| ***PMM2*** \| \| *PPA1* \| \| *PPA2* \| \| *PPP6C* \| | \| *PRSS23* \| \| --- \| \| *PSMB10* \| \| *PSMB5* \| \| *PSMB6* \| \| *PSMB7* \| \| *PSMB8* \| \| *PSMB9* \| \| *PSMD14* \| \| *QARS1* \| \| *QSOX1* \| \| *RAB10* \| \| *RAB11A* \| \| *RAB11B* \| \| ***RAB1A*** \| \| ***RAB1B*** \| \| *RAB27A* \| \| *RAB27B* \| \| *RAB5A* \| \| *RAB5B* \| \| *RAB5C* \| \| *RAB7A* \| \| *RABGGTA* \| \| *RABGGTB* \| \| *RARS1* \| \| *RBX1* \| \| *RHOA* \| \| *RIPK1* \| \| *RPS3* \| \| *RUVBL1* \| \| *SARS1* \| \| *SENP5* \| \| *ST3GAL1* \| \| *ST3GAL4* \| \| *ST3GAL6* \| \| *ST6GAL1* \| | \| ***ST6GALNA6*** \| \| --- \| \| *TARS1* \| \| *TPST2* \| \| *TRAF3* \| \| *TRIM25* \| \| *TTLL13* \| \| ***UAP1*** \| \| *UBA1* \| \| *UBA3* \| \| *UBA6* \| \| *UBE2D2* \| \| *UBE2D3* \| \| *UBE2I* \| \| *UBE2K* \| \| *UBE2L3* \| \| *UBE2L6* \| \| *UBE2M* \| \| *UBE2N* \| \| *UCHL3* \| \| *UCHL5* \| \| ***UGGT1*** \| \| ***UGGT2*** \| \| *USP14* \| \| *USP15* \| \| *USP24* \| \| *USP47* \| \| *USP5* \| \| *USP9X* \| \| *VARS1* \| \| ***VCP*** \| \| *WARS1* \| \| *YARS1* \| \| *YOD1* \| |
| Carbohydrate metabolism (91) | \| *AGL* \| \| --- \| \| *AKR1A1* \| \| *AKR1B1* \| \| *ALDH1A1* \| \| *ALDOA* \| \| *ALDOC* \| \| ***B3GAT3*** \| \| ***B3GNT2*** \| \| ***B3GNT3*** \| \| ***B3GNT7*** \| \| ***B4GALT1*** \| \| ***B4GAT1*** \| \| ***CHST1*** \| \| *CRYL1* \| \| *DCXR* \| \| *DERA* \| \| *ENO1* \| \| *ENO2* \| \| *ENO3* \| | \| ***EXT1*** \| \| --- \| \| *FBP1* \| \| *FBP2* \| \| ***FUT3*** \| \| ***FUT5*** \| \| ***FUT6*** \| \| *G6PD* \| \| *GAA* \| \| *GALE* \| \| *GALK1* \| \| *GALT* \| \| *GAPDH* \| \| *GAPDHS* \| \| *GBE1* \| \| *GLB1* \| \| *GNPDA1* \| \| *GNPDA2* \| \| *GOT1* \| \| *GOT2* \| | \| *GPI* \| \| --- \| \| *GYG1* \| \| ***HAS2*** \| \| ***HEXA*** \| \| ***HEXB*** \| \| *HK1* \| \| *HK3* \| \| ***IDS*** \| \| ***IDUA*** \| \| *KHK* \| \| *MAN2B1* \| \| *MAN2B2* \| \| *MAN2C1* \| \| *MANBA* \| \| *MDH1* \| \| *MDH2* \| \| *NAGLU* \| \| *NHLRC1* \| \| ***PAPSS1*** \| | \| *PFKL* \| \| --- \| \| *PFKM* \| \| *PFKP* \| \| *PGAM1* \| \| *PGD* \| \| *PGK1* \| \| *PGLS* \| \| *PGM1* \| \| *PGM2* \| \| *PGM2L1* \| \| *PKM* \| \| *PPP2CA* \| \| *PPP2CB* \| \| *PRPS1L1* \| \| *PRPS2* \| \| *PYGB* \| \| *PYGL* \| \| *PYGM* \| \| *RPE* \| | \| *RPIA* \| \| --- \| \| ***SGSH*** \| \| *SHPK* \| \| *SORD* \| \| ***ST3GAL1*** \| \| ***ST3GAL4*** \| \| ***ST3GAL6*** \| \| ***ST6GALNAC6*** \| \| *TALDO1* \| \| *TKFC* \| \| *TKT* \| \| *TPI1* \| \| *UGP2* \| \| ***XYLT1*** \| \| ***XYLT2*** \| |
| Lipid metabolism (82) | \| *ACADVL* \| \| --- \| \| *ACAT1* \| \| *ACAT2* \| \| *ACLY* \| \| *ACOX1* \| \| *AKR1B1* \| \| *AKR1C1* \| \| *AKR1C2* \| \| *AKR1C3* \| \| *AKR1D1* \| \| *ALDH3A2* \| \| *ALOX12B* \| \| *ARSA* \| \| *ASAH1* \| \| *BDH2* \| \| *CBR1* \| \| *CHKB* \| | \| *CRAT* \| \| --- \| \| *CTSA* \| \| *DECR1* \| \| *ECHS1* \| \| *EPHX2* \| \| *FAAH2* \| \| *FASN* \| \| *FDPS* \| \| *GBA* \| \| *GDPD3* \| \| *GGPS1* \| \| *GK* \| \| *GLA* \| \| *GLB1* \| \| *GPD1L* \| \| *GPX1* \| \| *GPX4* \| | \| *HADHA* \| \| --- \| \| *HADHB* \| \| ***HEXA*** \| \| ***HEXB*** \| \| *HMGCS1* \| \| *HSD17B4* \| \| *IDI1* \| \| *INPP4B* \| \| *KDSR* \| \| *LGMN* \| \| *LIPH* \| \| *LIPI* \| \| *LPIN3* \| \| *LTA4H* \| \| *ME1* \| \| *MGLL* \| \| *MOGAT1* \| | \| *MVK* \| \| --- \| \| *NEU1* \| \| *NEU2* \| \| *PCYT2* \| \| *PIP4K2C* \| \| *PLA2G15* \| \| *PLA2G2A* \| \| *PLA2G4B* \| \| *PLA2G4D* \| \| *PLA2G4E* \| \| *PLBD1* \| \| *PON1* \| \| *PPM1L* \| \| *PPP1CA* \| \| *PPP1CB* \| \| *PPP1CC* \| \| *PPT1* \| | \| *PTGDS* \| \| --- \| \| *PTGES* \| \| *PTGES3* \| \| *PTGR1* \| \| *PTGR2* \| \| *PTPN13* \| \| *RAB5A* \| \| *SCP2* \| \| *SGPP1* \| \| *SMPD1* \| \| *SULT2A1* \| \| *TECR* \| \| *TXNRD1* \| \| *UBE2I* \| |

**Table S3**

| (a)  Biological processes | | | | | |
| --- | --- | --- | --- | --- | --- |
| GO ID | **GO Term** | **Number** | **%** | **Log10(P)** | **Log10(q)** |
| GO:0005975 | Carbohydrate metabolic process | 132 | 14.7 | -91.93 | -87.58 |
| GO:0055086 | Nucleobase-containing small molecule metabolic process | 140 | 15.59 | -90.68 | -86.63 |
| GO:0044282 | Small molecule catabolic process | 93 | 10.36 | -60.14 | -56.64 |
| GO:0043603 | Cellular amide metabolic process | 132 | 14.7 | -59.67 | -56.22 |
| GO:0032787 | Monocarboxylic acid metabolic process | 104 | 11.58 | -57.03 | -53.68 |
| GO:0006790 | Sulfur compound metabolic process | 86 | 9.58 | -54.32 | -51.05 |
| GO:1901137 | Carbohydrate derivative biosynthetic process | 108 | 12.03 | -53.06 | -49.86 |
| GO:0044283 | Small molecule biosynthetic process | 86 | 9.58 | -45 | -41.95 |
| GO:1901615 | Organic hydroxy compound metabolic process | 87 | 9.69 | -42.77 | -39.75 |
| GO:0016052 | Carbohydrate catabolic process | 45 | 5.01 | -40.81 | -37.83 |
| GO:1901136 | Carbohydrate derivative catabolic process | 53 | 5.9 | -38.36 | -35.45 |
| GO:0044262 | Cellular carbohydrate metabolic process | 50 | 5.57 | -35.97 | -33.07 |
| GO:0006575 | Cellular modified amino acid metabolic process | 50 | 5.57 | -33.62 | -30.82 |
| GO:0010035 | Response to inorganic substance | 78 | 8.69 | -31.59 | -28.85 |
| GO:1901657 | Glycosyl compound metabolic process | 33 | 3.67 | -28.28 | -25.69 |
| (b)  Pathways | | | | | |
| Pathway ID | **Pathway Description** | **Number** | **%** | **Log10(P)** | **Log10(q)** |
| R-HSA-6798695 | Neutrophil degranulation | 107 | 11.92 | -60.99 | -57.42 |
| hsa01200 | Carbon metabolism | 59 | 6.57 | -58.1 | -54.71 |
| WP3925 | Amino acid metabolism | 39 | 4.34 | -34.62 | -31.79 |
| hsa01240 | Biosynthesis of cofactors | 45 | 5.01 | -31.39 | -28.66 |
| hsa00520 | Amino sugar and nucleotide sugar metabolism | 28 | 3.12 | -29.59 | -26.93 |
